# How Much Do We Know about Oral Cancer?—An Online Survey †

**DOI:** 10.3390/dj11120268

**Published:** 2023-11-24

**Authors:** Iva Horvat, Božana Lončar-Brzak, Ana Andabak Rogulj, Livia Cigić, Sonja Pezelj Ribarić, Miroslav Sikora, Danica Vidović-Juras

**Affiliations:** 1 Private Dental Clinic Zagreb, 10000 Zagreb, Croatia; horvativaa@gmail.com; 2 Department of Oral Medicine, School of Dental Medicine, University of Zagreb, 10000 Zagreb, Croatia; loncar@sfzg.hr (B.L.-B.);; 3 Croatian Society for Oral Medicine and Pathology, Croatian Medical Association, 10000 Zagreb, Croatia; 4 Croatian Association of Hospital Dentistry/Special Care Dentistry, Croatian Medical Association, 10000 Zagreb, Croatia; 5 Department of Oral Diseases, University Dental Clinic, University Hospital Centre Zagreb, 10000 Zagreb, Croatia; 6 Department of Oral Medicine and Periodontology, School of Medicine, University of Split, 21000 Split, Croatia; 7 Department of Oral Medicine, Faculty of Dental Medicine, University of Rijeka, Krešimirova 40, 51000 Rijeka, Croatia; 8 Department of Dental Medicine, Faculty of Dental Medicine and Health, Josip Juraj Strossmayer University of Osijek, Crkvena 21, 31000 Osijek, Croatia; 9 Clinic of Dental Medicine, Clinical Hospital Center Rijeka, Krešimirova 40, 51000 Rijeka, Croatia; 10 School of Medicine, Josip Juraj Strossmayer University of Osijek, 31000 Osijek, Croatia; miro_jr@yahoo.com

**Keywords:** oral cancer knowledge, education, healthcare students, doctors

## Abstract

Introduction: Oral cancer (OC) is a disease with a high mortality rate due to its late recognition. Since the oral cavity is easily accessible for visual inspection, enabling early diagnosis, the education of healthcare workers about preventive oral examinations is critical. This research aimed to assess the level of participants’ OC knowledge, as well as to raise awareness about this diagnosis. Materials and methods: The research was conducted as an online survey among students of dental medicine, students of medicine, doctors of dental medicine and doctors of medicine. The questionnaire was designed solely for the purpose of this study and consisted of 29 questions. The first part of the questionnaire consisted of general questions about the participants, whereas the questions in the second part addressed their knowledge and attitudes towards OC. Results: The surveyed population comprised of 140 dental students, 105 medical students, 159 doctors of dental medicine and 100 medical doctors. The level of knowledge about OC among the participants is not yet satisfactory. The group of dental medicine students scored highest, while medical doctors showed the weakest knowledge. Conclusion: Additional education about OC for doctors of dental medicine and medical doctors is needed. This step will improve prevention and increase chances for early detection.

## 1. Introduction

Oral cancer (OC) is a malignant disease which comprises the cancer of the oral cavity, lip cancer and oropharyngeal cancer [1,2,3]. It is the 16th most common malignant tumor and the 15th most common cause of death across the world, with a frequency of 4/100,000, varying among countries, races and ethnicity groups.

The occurrence of OC rises with age and reaches its peak in people older than 60 years [4]. Lately, there has been a significant prevalence increase in people younger than 40. Some authors claim that OC occurring in younger people is more aggressive, tends to relapse locally and overall has a worse prognosis than OC of older age [5]. Most of the patients suffering from OC are men, but the ratio between men and women is not as significant as it had been earlier. Today, more women suffer from OC due to the change in habits in terms of increased alcohol consumption and tobacco smoking [6].

OC is a squamous cell carcinoma in over 90% of the cases. It is predominantly found in areas such as the floor of the mouth, the ventral and lateral sides of the tongue, the retromolar area and the area of palatal arches [7]. According to the World Health Organization, every alteration of oral mucosa that persists for two weeks after the irritant removal should undergo a biopsy. Biopsy is a gold standard for diagnosing OC [4,8,9,10].

The prognostic factor of outmost importance is the histopathological stage of the tumor at the time of diagnosis [4]. Early detection increases survival [10,11,12,13,14]. It is known that the 5-year survival rate in grade 1 and 2 tumors is about 80%, and in grade 3 and 4 tumors, it is less than 20% [15]. Besides the effect on survival, early diagnosis decreases the treatment cost and preserves patients’ quality of life. Since the oral cavity is easily accessible for visual inspection, revealing suspicious oral lesions, the education of healthcare workers about preventive oral examinations is very important. Oral examinations take less than 2 min but can detect most OCs or mucosal alterations that can become OC [16]. Doctors of dental medicine, due to the nature of their work, have an excellent opportunity to perform routine oral examinations. However, sometimes, the patient will first see their family physician with oral complaints [17]. Therefore, it is essential that both dental and medical students and dentists and medical doctors are educated on this diagnosis.

Some studies worldwide have evaluated oral cancer awareness and practices among future and working dentists and medical students [18,19,20,21,22,23,24,25,26,27,28,29,30,31,32,33], showing different levels of knowledge and suggesting improvements in teaching.

This research aimed to obtain and analyze the data about OC knowledge among dental students, medical students, dentists and medical doctors from four cities in Croatia as well as to compare the results with previous similar studies regarding this subject. The results would show whether undergraduate teaching gives an adequate level of knowledge to students and whether the working knowledge of dental and medical clinicians is satisfactory. Our goal was also to raise awareness about OC among the participants and to motivate and educate them about the prevention and early recognition of this potentially fatal disease.

## 2. Materials and Methods

The research was conducted via an online survey questionnaire and was approved by the Ethics Committee of the University of Zagreb, School of Dental Medicine (approval number: 05-PA-30-VI-3/2022). The results are reported in accordance with the CHERRIES guidelines [34]. The questionnaire was designed for the purpose of this research and was neither validated nor piloted before distribution. An oral medicine specialist approved its content validity. The link for the survey was sent via e-mail to 800 addresses of fourth-, fifth- and sixth-year students of dental medicine and medicine and practicing dentists and doctors of medicine in the Republic of Croatia from our database. The link was available online for one month, and after that, the data were analyzed. Sample size calculation was not performed. Participation in the survey was anonymous and voluntary as stated in the cover letter accompanying the questionnaire. The participants completed an informed consent prior to participation. No incentive was offered to the participants for participation in the survey. An overall completion rate was 63% (504/800). The surveyed population comprised of 140 dental students, 105 medical students, 159 dentists and 100 medical doctors from the four largest cities in Croatia (Zagreb, Rijeka, Split and Osijek). Four respective questionnaires designed for this study evaluated the OC knowledge of the groups. Each questionnaire consisted of 29 close-ended questions displayed over four pages, and the responses were captured automatically. The same set of OC knowledge questions was the base of all questionnaires, with an addition of group-related personal-type questions. Collected data were statistically analyzed. Data were entered into the statistical software Med Calc 19.7.2 (MedCalc Software, Ostende, Belgium) and analyzed using descriptive statistics.

## 3. Results

The number of respondents and the percentage in the total number of each group of respondents is shown in Table 1.

Average university scores of dental and medical students in different cities in Croatia are shown in Table S1 in the Supplementary Materials. Overall, 82.1% and 87.6% of dental and medical students, respectively, were in their sixth and fifth years of study. Among dentists, 88.7% are general practitioners, while the rest are different specialists (11.3%). Regarding their working experience, 37.1% had graduated more than 20 years ago, 16.4% had graduated 15–20 years ago, 18.2% had graduated 10–15 years ago, 14.5% had graduated 5–10 years ago and 13.8% had graduated less than five years ago.

Among doctors of medicine, 49% of them are general practitioners, while the rest are different specialists. Regarding their working experience, 41% had graduated more than 20 years ago, 10% had graduated 15–20 years ago, 6% had graduated 10–15 years ago, 11% had graduated 5–10 years ago and 32% had graduated less than five years ago.

When asked whether they have learned about OC during their college education, 71.4% of medical students, 80.7% of dental students, 70% of medical doctors and 69.2% of dentists stated they have, but their confidence that they can recognize it was generally low. Only 53.3% of medical students, 62.1% of dental students, 59% of medical doctors and 64.8% of dentists believed they could recognize OC. Among the dental students, those from Zagreb were the most certain in their ability to recognize OC—67.7% of them chose the answer “Yes” compared to 61.7% of dental students in Rijeka and 56.7% of dental students in Split. The percentage of medical students from Split that chose “Yes” for an answer was 64.3%, compared to 54.8% of medical students in Zagreb, 56.4% of medical students in Rijeka and 20% of medical students in Osijek. Most of the examined medical students in Osijek chose the answer “I don’t know” (70%).

Only 37.14% of dental students, 46.67% of medical students, 59.75% of dentists and 8% of medical doctors have stated that they perform preventive oral examinations in every patient they see (Table S2, Supplement).

The correct answers to the following questions about the preventive oral examinations were as follows: it is non-invasive, it lasts less than 2 min, it does not require any of the extra equipment, it is not uncomfortable for the patient, it can be used to recognize cancer in the earliest stage, each person should be examined at least once a year, and it should include palpation of the lymph nodes of the neck. The percentage of participants who have had all answers correct is shown in Table 2.

The questions that followed were to assess the knowledge of OC risk factors. Most of the participants (96.2% of medical students, 97% of medical doctors, 97.1% of dental students and 93.7% of dentists) agreed that smoking is the most common risk factor for the development of OC.

Moreover, participants had to select all the OC risk factors. Possible answers were tobacco smoking; alcohol consumption; sun exposure; nutrition lacking vitamins and minerals; diabetes; spicy food consumption; viral infections; bad oral hygiene; obesity; and consumption of salted, smoked foods. All ten answers are risk factors for OC. The results are shown in Table 3.

**Figure 1:**
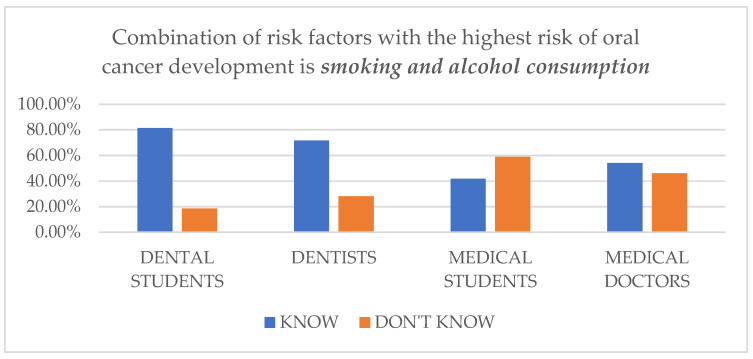
Respondents’ knowledge regarding the question of “Which of the following is the combination of risk factors with the highest risk of oral cancer development?”

In the next question, participants had to choose the combination of the two risk factors with the highest risk of OC development. The correct answer was smoking and alcohol consumption (Figure 1 and Table 4).

The latest question shows a noticeable difference between medical and dental professions. Only 41.9% of medical students and 54% of medical doctors, in contrast to 81.4% of dental students and 71.7% of dentists, chose the correct answer of smoking and alcohol consumption. Most of the medical students (51.4%) and a significant number of medical doctors (45%) believe that the combination of risk factors with the highest risk of OC development is smoking and HPV infection.

The participants had to choose the following incorrect statement: HPV infection is one of the most common risk factors for the development of OC of the floor of the mouth. Only 24.8% of medical students and 37% of medical doctors did so; the rest of them chose that the statement was correct. Somewhat better were dental students and dentists, 64.3% and 53.5% of them chose the statement that was incorrect.

**Figure 2:**
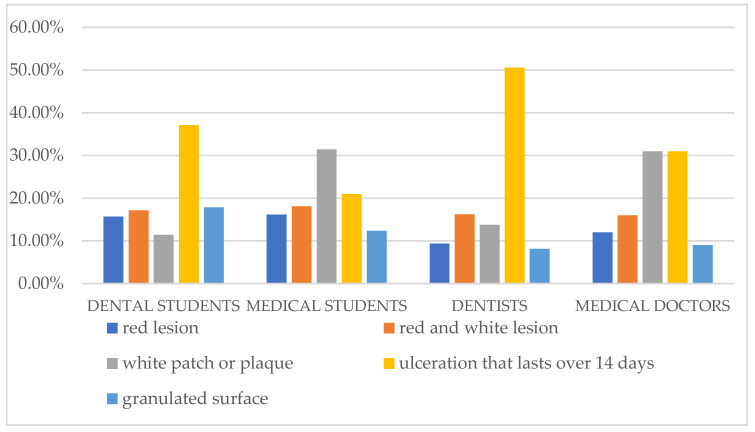
Participants’ answers about the earliest manifestation of oral cancer.

The most common answers among our respondents were ulceration lasting more than 14 days and a white patch or plaque. The participants were the least familiar with the granulated surface area on the oral mucosa as an early manifestation of OC (Figure 2).

Most of the respondents showed a good knowledge of OC histology. Overall, 87.6% of medical students, 81% of medical doctors, 92.1% of dental students and 71.7% of dentists knew that OC is mostly a squamous cell carcinoma. However, 10.5% of medical students, 12% of medical doctors, 2.9% of dental students and 10.1% of dentists believed that it was adenocarcinoma, and 1.9% of medical students, 7% of medical doctors, 5% of dental students and 18.9% of dentists thought that it was basal cell carcinoma.

The knowledge of different groups of participants and students in different cities about the most common localizations of OC is shown in Table 5 and Table 6.

The next question was the localization of oral cancer with the worst prognosis (the tongue), and this was answered correctly by 71% of dental students, 62% of dentists, 58% of medical doctors and only 40% of medical students. Knowledge differences among dental and medical students in different cities are shown in Figure S1 in the Supplementary Materials.

The participants in this study did not show much knowledge about OPMD, especially regarding the latest classification [35] (Table 7).

The most frequent responses in all groups of participants were oral lichen planus, erythroplakia and non-homogeneous leukoplakia.

When asked “*Which of the following conditions on the oral mucosa is already an OC in more than 50% of the cases?*”, dental students showed the most knowledge—77.9% of them answered this question correctly (they chose erythroplakia, among other offered answers of leukoplakia, oral lichen planus and pemphigoid). Furthermore, only 54.3% of medical students, 46.5% of dentists and 27% of medical doctors chose erythroplakia. The majority of medical doctors, 64% of them, incorrectly chose leukoplakia as their answer.

**Figure 3:**
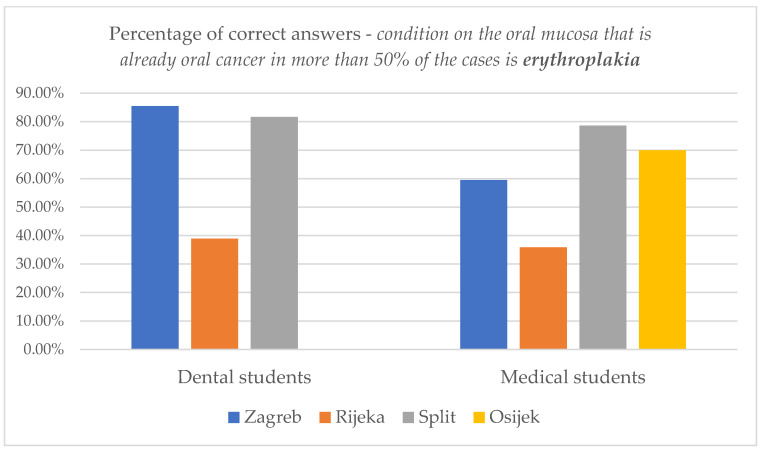
Knowledge differences about *the condition on the oral mucosa that is already oral cancer in more than 50% of the cases* in dental and medical students studying in different cities.

Differences in knowledge among dental and medical students studying in different cities on this issue are shown in Figure 3.

OC rarely has any symptoms in its earliest stages. Overall, 90% of dental students, 88.7% of dentists, 79% of medical students and 79% of medical doctors knew that. The rest chose one of the following symptoms as the leading symptom in the early stage of OC: halitosis, pain, bleeding, trismus, difficulty swallowing, excessive drooling, or swelling. This is incorrect, as all of those occur in the advanced stage of OC. The results showing the differences in knowledge about the symptomatology of OC are presented in the Supplementary Materials (Tables S3 and S4 and Figure S6).

Students (dental and medical) showed the best knowledge on the last question, almost 50% of them selected all six of the possible OC symptoms (discomfort and pain in the oral cavity, bleeding, difficulty chewing or swallowing, loss of sensation in part of the face, excessive mobility of the teeth and the enlargement of the lymph nodes in the neck). The symptoms most familiar to the participants were discomfort and pain in the oral cavity, bleeding and the enlargement of the lymph nodes in the neck. On the other hand, symptoms that are not so well known are excessive mobility of the teeth and loss of sensation in part of the face.

The answers of different groups of participants to the question about the survival rate of OC are shown in Table S5 in the Supplementary Materials. Differences in the knowledge of students in different cities regarding the prognosis of OC are shown in Table S4 in the Supplementary Materials.

The correct answer to this question is a malignant tumor with a high mortality rate. More than half of our respondents chose this answer (64.8% of medical students, 55% of medical doctors, 86.4% of dental students and 57.9% of dentists). Students of dental medicine showed the best knowledge, especially those studying in Zagreb.

Most medical students and medical doctors would refer a patient with a suspicious lesion to a maxillofacial surgeon (90.5% of medical students and 81% of medical doctors), otorhinolaryngologist (44.8% of medical students and 60% of medical doctors) or finally to an oral medicine specialist (50.5% of medical students and 46% of medical doctors). On the other hand, the majority of dental students and dentists would choose an oral medicine specialist (93.6% of dental students and 86.2% of dentists).

When asked about attending a professional course on OC in the last five years, most of the participants answered negatively (Table 8), but they stated that they would like to learn more (Table 9).

The number of students who answered all questions correctly was analyzed (Table 4, Table 6, Tables S3 and S6 in the Supplementary Materials, Figure 3 in the manuscript and Figure S1 from the Supplementary Materials). Overall, 27.1% (38/140) of dental students and only 1.9% (2/105) of medical students answered each question correctly. Dental students studying in Zagreb scored the best, followed by dental students from Split and Rijeka, as shown in the table below (Table 10). Most of these students (76.3%) had an average university score above 4.0 (Table S7 in the Supplementary Materials). In the category of medical students, only two answered each of the selected questions correctly: one student from Zagreb and one from Rijeka.

## 4. Discussion

A detailed examination of the oral cavity plays a key role in establishing an early diagnosis and lowering the mortality rate from OC. However, many healthcare professionals fail to do so. Experts agree that dentists or medical doctors do not need to know how to diagnose OC but need to recognize any suspicious changes in the oral mucosa and then refer the patient to the right specialist [17,36,37]. Results from the literature regarding oral cancer knowledge are variable. Kujan et al. [23]. showed poor results in assessing oral cancer knowledge among undergraduate medical students, where 72% of participants declared that they did not feel confident enough to perform an oral examination. A recent study that evaluated oral cancer knowledge among international dental and medical students at the Lithuanian University of Health Sciences [19] showed that half of the participants (56.92%) had fairly good knowledge of oral cancer risk factors, while almost one-third (28.06%) had poor knowledge. The score for international medical students was higher than that for dental students. Many studies have also shown low oral cancer awareness among dental students [18,19,20,24,25,26,27,28,29,30,31,38,39,40] and dentists [41,42,43]. The results of the present study showed that the percentage of doctors who perform preventive oral examinations in practice is very low, in contrast to students (46.7% of medical students and only 8% of doctors of medicine). On the other hand, it seems that dentists perform examinations more often after graduation (from 37.1% of students to 59.7% of dentists). These results are better than 6% of medical doctors and 53% of dentists in the previous 2010 research [44]. In another research from 2019, 65.6% of examined dentists perform preventive oral examinations [45]. The increase in the number of healthcare professionals performing oral examinations in recent studies is slight and lower than expected, and there is room for improvement.

Most of the participants in this study stated that they were taught about OC during their education. However, the percentage of those who truly think they can recognize it when they see it is smaller (53.3% of medical students, 62.1% of dental students, 59% of medical doctors and 64.8% of dentists). Only 45.7% of dental students, 44.76% of medical students, 20.76% of dentists and 25% of medical doctors chose all six mentioned symptoms of OC (pain and discomfort in the oral area, bleeding, increase in neck lymph nodes, difficulties swallowing, loss of sensation in part of the face and increasing teeth mobility). In the research from Žaja in 2010 only 11% of dentists and 3% of medical doctors chose the symptoms correctly [44]. There is improvement, but it is still unsatisfactory. The question arises as to how to improve the diagnosis of OC when competent experts cannot recognize the earliest manifestations of oral cancer and all possible symptoms.

Only 53.57% of dental students, 32.7% of dentists, 24.76% of medical students and 8% of medical doctors knew all three of the most common localizations of OC—the floor of the mouth, retromolar area, and ventral and lateral areas of the tongue. The localization, which most of the participants are familiar with, is the floor of the mouth. Participants in the present study showed poorer knowledge compared to the 2010 research [44], where 65% of dentists and 25% of medical doctors chose the correct answers, and similar knowledge as the participants in a research in 2019 [45]—39.3% of dentists. According to these results, participants in more recent studies showed worse knowledge than in the study from 2010. Out of the listed localizations (tongue, buccal mucosa, gingiva, hard palate, lips, and all of the above), cancer of the tongue has the worst prognosis. Only 40% of medical students, 56% of medical doctors, 70.7% of dental students and 63.5% of dentists answered correctly. Once again, dental students showed the best knowledge, while on the other hand, a great percentage of medical students (41%) and medical doctors (36%) thought that localization with the worst prognosis is hard palate or all of the above. Studies from 2016 [7] and 2019 [45] showed that dental students also chose the tongue as the highest-risk localization—80.8% of them in the latter study. The percentage of medical students that chose tongue was 35.4%. Moreover, 28.3% of them chose hard palate, comparable to 22.9% of medical students in the present research.

Knowing and recognizing risk factors for OC is of utmost importance for healthcare professionals so they can educate their patients and encourage them to avoid those risk factors as much as possible. Also, dentists and medical doctors must recognize and pay more attention to patients with a higher risk of OC.

In the present research, as well as in the research of Carter and al. [8], dental and medical students showed good knowledge about the biggest risk factors for OC. In both studies, most of the examined students have chosen correct answers (smoking tobacco and alcohol consumption). Those were also mostly chosen answers in a different study among dentists [17].

The classification of different oral conditions, so-called oral potentially malignant disorders (OPMDs), was updated in 2020 [16]. Before that, it included bullous lesions which are no longer part of the classification due to a lack of evidence about their malignant transformation. None of the participants in the present study chose all of the OPMDs correctly, indicating they are not familiar with the new updated guidelines. The most common answers were erythroplakia and non-homogeneous leukoplakia, which correlates with the study of Carter and al. [17]. The examined dental and medical students in the present research showed similar knowledge in the OPMD area, while in the research stated above (Carter et al.—conducted in the United Kingdom, 2007) medical students did much worse than dental students. Present results showed that 80% of medical students chose erythroplakia and 86.7% of them chose non-homogenous leukoplakia, compared to 21% and 34% in the research of Carter and al. [17].

OC prognosis is directly correlated with the stage of cancer at the time of detection. Chances of survival are much higher if the cancer is still in its earliest stages. Sadly, most OCs are diagnosed too late, with low chances. Our participants are aware of that; overall, 86.4% of dental students, 64.8% of medical students, 57.9% of dentists and 55% of medical doctors knew that in more than 50% of the cases, OC is in its advanced stage at the time of diagnosis. The fact that OC is a malignant tumor with a high mortality rate is known to 64.3% of dental students, 64.8% of medical students, 56% of dentists and 59% of medical doctors. However, a great percentage of the participants in the present study still think that it is a malignant tumor with a high survival rate. This is concerning because it shows their complete attitude towards OC is incorrect because they do not find it as dangerous as it should be perceived. Consequently, fewer doctors are doing preventive oral examinations, which prolongs the diagnosis of OC. There are no previously published studies to compare the answers to this question.

The majority of examined dental students (93.6%) and dentists (86.2%) would instruct a patient with a suspicious oral lesion to an oral medicine specialist, which corresponds with previous research [45] in which 81.5% of dental students would also instruct a patient to the mentioned specialist. On the other hand, the majority of medical students and medical doctors would refer the patient to a maxillofacial surgeon (90.5% of medical students and 81% of medical doctors), otolaryngologist (44.8% of medical students and 60% of medical doctors) or, finally, a specialist in oral medicine (50.5% of medical students and 46% of medical doctors). In the research mentioned, most medical students (45.8%) would choose an otolaryngologist, and only 28.3% would choose an oral medicine specialist [46].

The participant group that scored the highest was dental students, as expected. Many of them have recently learned about OC so the information is still fresh, and they are more familiar with the topic than medical students. This corresponds with previous studies [45,46,47]. Dentists showed greater knowledge between medical and dental professions, the same as in previous similar studies on the subject [44,47,48].

The knowledge of OC remains unsatisfactory, particularly because the quality of life of many patients depends on it. Despite the lack of knowledge, most participants have not attended a single educational professional course with the topic of OC in the last five years, which corresponds with the previous study [44].

However, as in other studies [44,48], most participants stated they would like to know more about OC. Improving knowledge about oral cancer would lead to the earlier referral of patients to a specialist and earlier treatment, increasing survival.

The strength of the present study is the side-by-side comparison of four different groups of participants regarding their knowledge of OC. These four groups of participants are future and current healthcare workers, which are in the closest contact with the general population and therefore are key points for improving prevention. Another advantage of the study is the relatively large number of participants and fair questionnaire completion rate. On the other hand, the limitation of the study is the limited number of participants in different cities due to voluntary participation. Perhaps the topic was not so attractive for all the invited participants, or the participants have not had time for time-consuming surveys. Also, the survey was not validated, but an oral medicine specialist approved its content validity. In our future research, we will try to use a validated questionnaire because it increases the research quality.

## 5. Conclusions

It is necessary to raise awareness about this disease in the general population but also in students and doctors of medicine and dental medicine to improve the prevention, recognition and the timely diagnosis of OC. All groups of participants have declared they would like to learn more about this topic. Among these four groups, dental students have shown the best knowledge about OC. Nevertheless, some areas could be improved during the undergraduate education of dental and medical students. As expected, dentists have shown greater knowledge about this topic than doctors of medicine, probably due to their nature of work and more focused education. The topic of OC should be represented in postgraduate education and in lifetime education professional courses to increase its awareness among practicing doctors of dental medicine and medical doctors.

## Figures and Tables

**Table 1:** Number and percentage of respondents in four selected cities in Croatia.

	Total	Zagreb		Rijeka		Split		Osijek	
	N	N	%	N	%	N	%	N	%
Dental students	140	62	44.3	18	12.9	60	42.9	/	/
Medical students	105	42	40	39	37.1	14	13.3	10	9.5
Dentists	159	123	76.9	32	20	3	1.9	/	/
Medical doctors	100	64	64	31	31	2	2	2	2

*N*—Number of respondents, %—percentage of respondents.

**Table 2:** Participants’ knowledge on the question about preventive oral examinations.

	Percentage of Those Who Selected All the Correct Answers
Dental students	65.7%
Medical students	54.3%
Medical doctors	40%
Dentists	43.4%

**Table 3:** Answers to the question about oral cancer risk factors.

	Selected All 10 of the Risk Factors	Selected “Smoking”	Selected Both “Smoking” and “Alcohol Consumption”	Selected “Smoking, Alcohol Consumption” and “Sun Exposure"
Dental students	4.29% (6)	100%	99.3%	34.3%
Dentists	1.89% (3)	100%	97.5%	31.25%
Medical students	13.33% (14)	100%	98.1%	32.4%
Medical doctors	12% (12)	100%	98%	25%

**Table 4:** Knowledge differences between dental and medical students studying in different cities regarding the question of “Which of the following is the combination of risk factors with the highest risk of oral cancer development?”.

	Dental Students			Medical Students			
	Zagreb	Rijeka	Split	Zagreb	Rijeka	Split	Osijek
Answered correctly	88.7%	83.3%	73.3%	38.1%	41%	50%	50%

**Table 5:** Participants’ knowledge of the three most common localization sites of oral cancer (the floor of the mouth, retromolar area, and ventral and lateral areas of the tongue).

	Answered Correctly	Answered Incorrectly
Dental students	53.57%	46.43%
Dentists	32.7%	67.3%
Medical students	24.76%	75.24%
Medical doctors	8%	92%

**Table 6:** Differences in knowledge about the most common sites of localization of oral cavity cancer among dental and medical students studying in different cities.

		Answered Correctly
Dental students	Zagreb	53.2% (33/62)
	Rijeka	44.4% (8/18)
	Split	56.7% (34/60)
Medical students	Zagreb	26.2% (11/42)
	Rijeka	25.6% (10/39)
	Split	21.4% (3/14)
	Osijek	20% (2/10)

**Table 7:** Participants’ responses to the task of Choose all the OPMD according to the most recent classification.

	Selected All the Correct Answers	Selected Only Correct Answers, but Not All of Them	Selected Some of the Incorrect Answers
Dental students	0	64.29%	35.71%
Dentists	0	55.45%	44.65%
Medical students	0	52.38%	47.62%
Medical doctors	0	43%	57%

**Table 8:** Answers to the question of Have you attended any of the professional courses on oral cancer in the last 5 years?

	No, There Wasn’t a Course Like That.	No, I Think I Know Enough about the Subject.	No, I’m Not Interested in the Subject.	Yes.
Dental students	63.3%	7.1%	2.1%	21.4%
Dentists	64.8%	3.8%	6.9%	24.5%
Medical students	67.6%	3.8%	24.8%	3.8%
Medical doctors	75%	1%	19%	5%

**Table 9:** Answers to the question of Would you like to learn more about oral cancer?

	Yes.	No.
Dental students	96.4%	3.6%
Dentists	93.1%	6.9%
Medical students	77.1%	22.9%
Medical doctors	83%	17%

**Table 10:** Differences in the knowledge of dental students studying in different cities.

	Chose All the Correct Answers
Zagreb	35.5% (22/62)
Rijeka	16.7% (3/18)
Split	21.7% (13/60)

## Data Availability

All relevant data are within the manuscript.

## Supplementary Materials

The following supporting information can be downloaded at: https://www.mdpi.com/article/10.3390/dj11120268/s1, Table S1: Average university scores of dental and medical students which have participated in the survey in different cities in Croatia; Table S2: Answers of the respondents to the following statement I’m doing the preventive oral examinations...; Figure S1: Knowledge differences regarding the localization site of oral cancer with the worst prognosis among dental and medical students studying in different cities; Table S3: Knowledge differences regarding the earliest oral cancer symptomatology in dental and medical students studying in different cities; Figure S2. Answers to the question regarding all the possible symptoms of oral cancer; Table S4: Participants’ knowledge on the question about all the possible oral cancer symptoms; Table S5: Answers to the question Oral cancer is….; Table S6: Knowledge differences regarding the oral cancer prognosis in dental and medical students studying in different cities (In the time of diagnosis of oral cancer…); Table S7: Average university scores of dental students who answered correctly to each of the chosen questions.
