# Supplementary material for: How Much Do We Know about Oral Cancer?—An Online Survey
Source: Dent J (Basel). 2023 Nov 24;11(12):268. doi: 10.3390/dj11120268 (PMC10743054; doi:10.3390/dj11120268)
Supplement: Supplementary file 1 [file dentistry-11-00268-s001.zip › dentistry-2620463-supplementary.docx]

Supplement

Tables and figures

Table S1. Average university scores of dental and medical students which have participated in the survey in different cities in Croatia.

|  | Dental students | | | Medical students | | | |
| --- | --- | --- | --- | --- | --- | --- | --- |
|  | Zagreb | Rijeka | Split | Zagreb | Rijeka | Split | Osijek |
| 2.0 – 3.49 | 1 | / | 4 | 2 | 2 | / | / |
| 3.5 – 4.0 | 12 | 6 | **32 (53.3%)** | 10 | 14 | **6 (42.9%)** | 3 |
| 4.1 – 4.49 | **34 (54.8%)** | **11 (61.1%)** | 20 | **18 (42.9%)** | **16 (41%)** | 5 | 3 |
| 4.5 – 5.0 | 15 | 1 | 4 | 12 | 7 | 3 | **4 (40%)** |

School score system in Croatia consists of 5 grades ( 1 – fail/not enough, 2 – below average/enough, 3 – average/good, 4 – above average/very good, 5 – excellent).

Table S2. Answers of the respondents to the following statement *I’m doing the preventive oral examinations...*

|  | *…in every patient I see.* | *…only in patients with higher risk.* | *…only if a patient is having symptoms.* | *…never* | *…only in patients in the Department of Oral Medicine.* (Only for dental students) |
| --- | --- | --- | --- | --- | --- |
| Dental students | 52 (37.14%) | 9 | 15 | 11 (7.9%) | 53 |
| Medical students | 49 (46.67%) | 16 | 36 | 4 (3.8%) | / |
| Dentists | 95 (59.75%) | 24 | 40 | 0 | / |
| Medical doctors | 8 (8%) | 19 | 65 | 8 (8%) | / |

Knowledge differences among dental and medical students in different cities are shown in Fig.1.


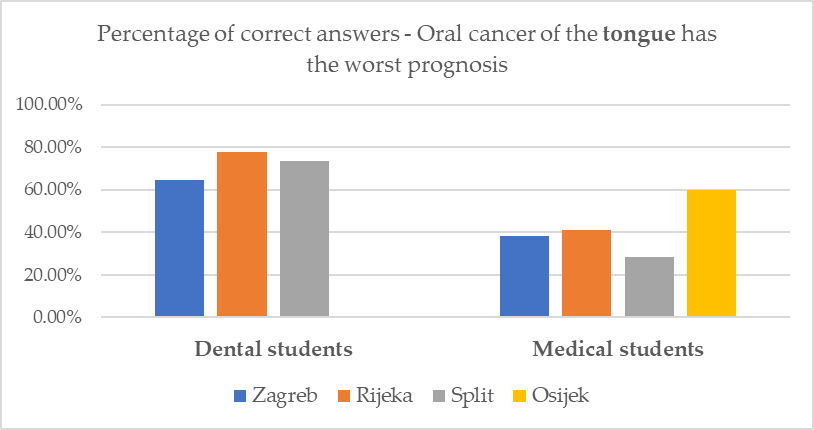


Figure S1. Knowledge differences regarding the localization site of oral cancer with the worst prognosis among dental and medical students studying in different cities.

Table S3. Knowledge differences regarding the earliest oral cancer symptomatology in dental and medical students studying in different cities.

|  | Dental students | | | Medical students | | | |
| --- | --- | --- | --- | --- | --- | --- | --- |
|  | Zagreb | Rijeka | Split | Zagreb | Rijeka | Split | Osijek |
| *Most likely there are no symptoms in the earliest stages of oral cancer.* | 98.4% (61/62) | 94.4% (17/18) | 81.7% (49/60) | 83.3% (35/42) | 74.4% (29/39) | 78.6% (11/14) | 80% (8/10) |


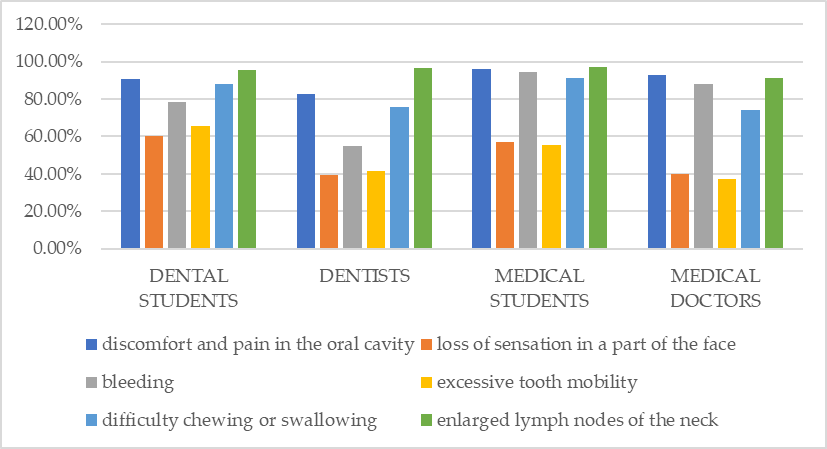


Figure S2. Answers to the question regarding all the possible symptoms of oral cancer.

Table S4. Participants’ knowledge on the question about all the possible oral cancer symptoms.

|  | Selected all 6 symptoms | 1 symptom is missing | 2 symptoms are missing | 3 or more symptoms are missing |
| --- | --- | --- | --- | --- |
| Dental students | 64 (45.7%) | 25 | 25 | 26 |
| Dentists | 33 (20.76%) | 22 | 30 | 74 |
| Medical students | 47 (44.76%) | 20 | 25 | 13 |
| Medical doctors | 25 (25%) | 17 | 26 | 32 |

Table S5. Answers to the question Oral cancer is….

|  | *…benign tumor with high survival rate.* | *…malignant tumor with high survival rate.* | *…malignant tumor with high mortality rate.* |
| --- | --- | --- | --- |
| Dental students | 1.4% | 34.3% | 64.3% |
| Dentists | - | 44% | 56% |
| Medical students | - | 35.2% | 64.8% |
| Medical doctors | - | 41% | 59% |

Table S6. Knowledge differences regarding the oral cancer prognosis in dental and medical students studying in different cities (In the time of diagnosis of oral cancer…).

|  | Dental students | | | Medical students | | | |
| --- | --- | --- | --- | --- | --- | --- | --- |
|  | Zagreb | Rijeka | Split | Zagreb | Rijeka | Split | Osijek |
| *…most of oral cancer cases are in the earliest stages.* | 0 | 0 | 1 | 1 | 1 | 0 | 0 |
| *… in over 20% of the cases oral cancer is in advanced stage.* | 3 | 3 | 11 | 9 | 14 | 5 | 5 |
| *…in over 50% of the cases oral cancer is in advanced stage.* | **59 (95.2%)** | **14 (77.8%)** | **48  (80%)** | **31 (73.8%)** | **23  (59%)** | **9 (64.3%)** | **5  (50%)** |
| *… the frequency of oral cancer cases in early and advanced stage is similar.* | 0 | 0 | 0 | 1 | 1 | 0 | 0 |
| *… stage of the tumor in the time of diagnosis doesn’t impact the prognosis of oral cancer.* | 0 | 1 | 0 | 0 | 0 | 0 | 0 |

Table S7. Average university scores of dental students who answered correctly to each of the chosen questions.

| **Average university score** | **Percentage** |
| --- | --- |
| 2.0 – 3.49 | 5.3% (2/38) |
| 3.5 – 4.0 | 18.4% (7/38) |
| 4.1 – 4.49 | 52.6% (20/38) |
| 4,5 – 5.0 | 23.7% (9/38) |
